# A New Species of *Murina* (Chiroptera: Vespertilionidae) from Yunnan, China †

**DOI:** 10.3390/ani14162371

**Published:** 2024-08-15

**Authors:** Xin Mou, Yishun Qian, Mei Li, Biao Li, Xiong Luo, Song Li

**Affiliations:** 1 Kunming Natural History Museum of Zoology, Kunming Institute of Zoology, Chinese Academy of Sciences, Kunming 650223, China; mouxin@mail.kiz.ac.cn (X.M.);; 2 Conservation Area Management Committee of Guanyin Shan Provincial Nature Reserve, Yuanyang 662400, China; 3 Yunnan Key Laboratory of Biodiversity Information, Kunming Institute of Zoology, Chinese Academy of Sciences, Kunming 650201, China

**Keywords:** *Murina*, new species, taxonomy, morphology, China, COI

## Abstract

**Simple Summary:**

In this paper, a new *Murina* species is described from Yunnan, China, based on morphological and molecular evidence. Genetically, the new species is most closely related to *M. chrysochaetes*. Morphologically, the new species can be distinguished from *M. chrysochaetes* and all other congeners by a combination of morphological characteristics. The discovery of the new species brings the total number of recognized species of the genus *Murina* to 42, of which 22 occur in China.

**Abstract:**

During an examination of various specimens previously collected from different locations and times, we discovered seven *Murina* specimens that had been collected in May 2023 from the Guanyinshan Provincial Nature Reserve, Yuanyang, Yunnan, China. Based on the assessment of morphological characteristics and molecular data analysis, these specimens were determined to represent a previously unidentified species, designated *Murina yuanyang* sp. nov.

## 1. Introduction

Members of the genus *Murina*, belonging to the subfamily Murininae within the family Vespertilionidae, are characterized by distinct tubular nostrils that extend laterally from the muzzle. In terms of dentition, they possess two well-developed upper and lower premolars, with a tendency for reduced complexity in molar structures [1]. Corbet and Hill [2] classified *Murina* into two groups, the ‘s*uilla*-group’ and ‘c*yclotis*-group’, based on the relative size of the crown area of canines, the first and second upper premolars, and the position of the incisors [2,3,4,5,6,7,8,9,10,11,12]. However, these two morphogroups do not represent separate phylogenetic lineages and will therefore be referred to hereafter as the ‘s*uilla*-type’ and the ‘*cyclotis*-type’. In contrast, the genus *Harpiocephalus* can be distinguished from *Murina* by a combination of body size (forearm length usually greater than 44.1 mm) and craniodental structure (the skull is more heavily built with the rostrum relatively shorter, broader, more abrupt and truncated anteriorly, and the third upper molar (M^3^) is highly reduced) [13]. *Harpiola* and *Murina* can also be distinguished based on the following diagnostic characteristics: the heights of the inner (I^2^) and outer upper incisors (I^3^) each measure two-thirds the height of the upper canines (C^1^) (vs. half in *Murina*); the height of the upper toothrow gradually decreases sequentially from C^1^ to the first premolar (P^2^) and then to the second premolar (P^4^), with each tooth maintaining similar volumes (vs. a noticeably smaller P^2^ compared to the other two teeth in *Murina*); the height and volume of the lower canine (C_1_), first premolar (P_2_), and second premolar (P_4_) are similar (vs. significantly smaller P_2_ than C_1_ in *Murina*); and C_1_ is strongly bifid with well-developed additional cusps (vs. very small secondary cingular cusps in *Murina*) [14,15].

Simmons [16] confirmed the presence of 17 species within the *Murina* genus. Since then, enhanced capture techniques and advances in molecular approaches have led to the description of various new species within the genus [1,4,5,6,7,8,9,10,11,12,14,17,18,19,20,21]. At present, 41 species are listed in the Global Biodiversity Information Facility (GBIF), with 21 species distributed in China according to the Catalogue of Mammals in China [22].

During an examination of various specimens previously collected from different locations and times, we identified seven *Murina* specimens collected in May 2023 from the Guanyinshan Provincial Nature Reserve, Yuanyang, Yunnan, China, all belonging to the family Vespertilionidae. Morphologically, the specimens exhibited external characteristics typical of the Murininae subfamily, including distinct tubular nostrils that are separated from each other and protrude laterally from the muzzle. Their smaller size, atypical for the genus *Harpiocephalus*, and distinct dental proportions (height of I^2^ and I^3^ less than half the height of C^1^ and height and size of P^2^ significantly smaller than C^1^ and P^4^), atypical for the genus *Harpiola*, suggested an affiliation with the genus *Murina*. However, their unique morphological features were inconsistent with any currently identified *Murina* species in China. Thus, based on morphological characteristics and molecular data, these specimens were identified as a new species, described herein as *Murina yuanyang* sp. nov.

## 2. Materials and Methods

### 2.1. Sample Collection

Specimens of *Murina yuanyang* sp. nov. include two adult males and five adult females, collected in the Guanyinshan Provincial Nature Reserve, Yuanyang, Yunnan, China, in May 2023. The voucher specimens are stored at the Kunming Natural History Museum of Zoology, Kunming Institute of Zoology, Chinese Academy of Sciences (KIZ, CAS), Kunming, China, under field collection numbers KIZ20230415, 20230424, 20230426, 20230449, 20230450, 20230492, and 20230498.

### 2.2. Measurements

External measurements were taken using a digital caliper accurate to 0.01 mm, measuring 10 indices, including head–body length (HB): from tip of snout to anus; tail length (TL): from anus to tip of tail; ear length (E): from lower edge of external auditory meatus to tip of pinna; hindfoot length (HF): from extremity of heel to tip of longest toe, not including claws; tibia length (TIB): from knee joint to ankle; forearm length (FA): from elbow to carpus with wings folded; and length of metatarsal of second, third, fourth, and fifth digits (MET2, MET3, MET4, and MET5, respectively): from carpus to end of respective metacarpals. Body weight (WT) was measured using an electronic scale accurate to 0.1 g.

Cranial and dental measurements were taken with a digital caliper to the nearest 0.01 mm under a stereomicroscope by Xin Mou in three independent measurements and finally averaged, with craniodental measurement definitions shown in Table 1.

### 2.3. Molecular Analyses

Total genomic DNA was extracted from muscle samples using the TSINGKE TSP202-50 Trelief ^®^ Hi-Pure Animal Genomic DNA Kit (Tsingke Biotech, Beijing, China) following the manufacturer’s protocols. The mitochondrial cytochrome oxidase subunit 1 (COI) gene sequence was amplified and sequenced using the following primer pair: BIF: TCAACCAACCACAAAGACATTGGCAC; BIR: TAGACTTCTGGGTGGCCAAAGAATCA. Polymerase chain reaction (PCR) was conducted in a total volume of 50 μL, including template DNA (1 μL), each primer (10 pM, 2 μL), and GOLD mix (Green-TSINGKE TSE101) (45 μL). The PCR procedure consisted of initial denaturation at 94 °C for 2 min, 5 cycles with denaturation at 94 °C for 30 s, annealing at 50 °C for 40 s, extension at 72 °C for 1 min, 35 cycles with denaturation at 94 °C for 30 s, annealing at 55 °C for 40 s, extension at 72 °C for 1 min, with a final extension at 72 °C for 10 min, and 4 °C for renaturation. The PCR products were analyzed by agarose gel electrophoresis and purified using the Trelief^®^ DNA Gel Extraction Kit (Tsingke Biotech, Beijing, China). Finally, purified samples were sequenced using an ABI 3730XL DNA Analyzer (USA) at Tsingke Biotech (Beijing, China). The sequencing files were checked and assembled using SeqMan in Lasergene v7.1 (DNASTAR Inc., Madison, WI, USA).

The COI sequences were compared to 32 subfamily Murininae species sequences downloaded from the National Center for Biotechnology Information (NCBI) database using PhyloSuite v1.2.2 [23], with their accession numbers listed in Table 2. All sequences were aligned using the ClustalW algorithm [24] with default parameters in MEGA11 [25] and were truncated to 657 bp. The uncorrected *p*-distances were calculated using the pairwise distance parameter in the distance module in MEGA11 with a bootstrap procedure of 1000 replicates, employing the pairwise deletion option to remove ambiguous positions. ModelFinder [26] was used to select the best-fit model based on Bayesian information criterion (BIC). Phylogenetic reconstruction was carried out using Bayesian inference (BI) under the GTR+F+I+G4 model with MrBayes v3.2.6 [27] in PhyloSuite v1.2.2 [24], employing a partition model with two parallel runs and 2,000,000 generations, discarding the initial 25% of sampled data as burn-in. Maximum-likelihood phylogenies were inferred using IQ-TREE [28] under the TPM2u+F+R3 model with 5000 ultrafast bootstraps [29] and the Shimodaira-Hasegawa-like approximate likelihood ratio test [30].

## 3. Results

### 3.1. Molecular Phylogenetic

We compared the COI sequences of six specimens (one was not sequenced) with those of 32 subfamily Murininae species (*Kerivoula kachinensis* and *Myotis muricola* were used as the outgroup), downloaded from the NCBI database. The species and their GenBank accession numbers are listed in Table 2. All novel sequences were deposited in the NCBI GenBank database under accession numbers PQ179688-179693.

**Figure 1:**
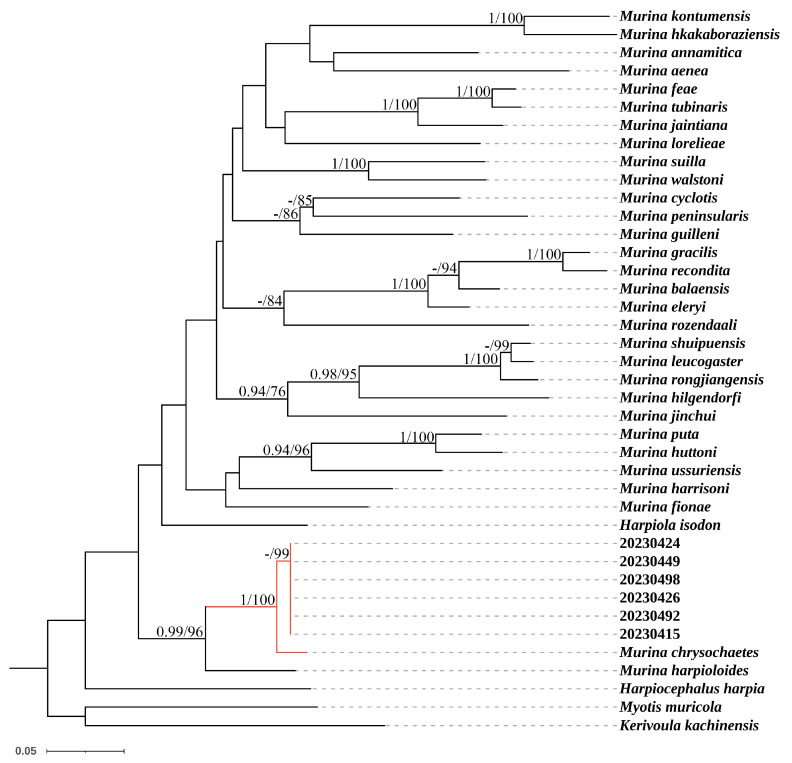
Bayesian phylogenetic tree of *Murina* species based on COI fragments. Node numbers before “/” indicate Bayesian posterior probabilities (values below 0.90 not shown) and numbers after “/” indicate ultrafast bootstrap support for maximum-likelihood analyses (values below 70 not shown).

The reconstructed phylogenetic tree revealed that all *Murina yuanyang* sp. nov. formed a clade and a distinct lineage sister to *M. chrysochaetes* with a posterior probability of 1 and bootstrap value of 100 (Figure 1). Uncorrected *p*-distances calculated between these two species ranged from 2.7% to 3.0% (Table 3).

### 3.2. Systematic Description

*Murina yuanyang* sp. nov.

Holotype: Field number KIZ20230424, adult female, collected on 21 May 2023. The mitochondrial COI nucleotide sequence was submitted to GenBank under accession number PQ179689.

Type locality: Pinghe, Xiaoxinjie Town, Yuanyang County, Yunnan Province, China (22.990097° N, 102.990097° E, 2434 m).

Paratype: Field number KIZ20230450, adult female, collected in Pinghe, Xiaoxinjie Township, Yuanyang County, Yunnan Province, China (22.990097° N, 102.990097° E, 2434 m) on 22 May 2023. Field number KIZ20230415, adult male, collected in Pinghe, Xiaoxinjie Township, Yuanyang County, Yunnan Province, China (22.991851° N, 103.002188° E, 2412 m) on 21 May 2023. The mitochondrial COI nucleotide sequence of KIZ20230415 was submitted to GenBank under accession number PQ179688.

Etymology: The name *yuanyang* refers to the type locality of the species.

Measurements: Measurements of the type specimen are shown in Table 4.

**Figure 2:**
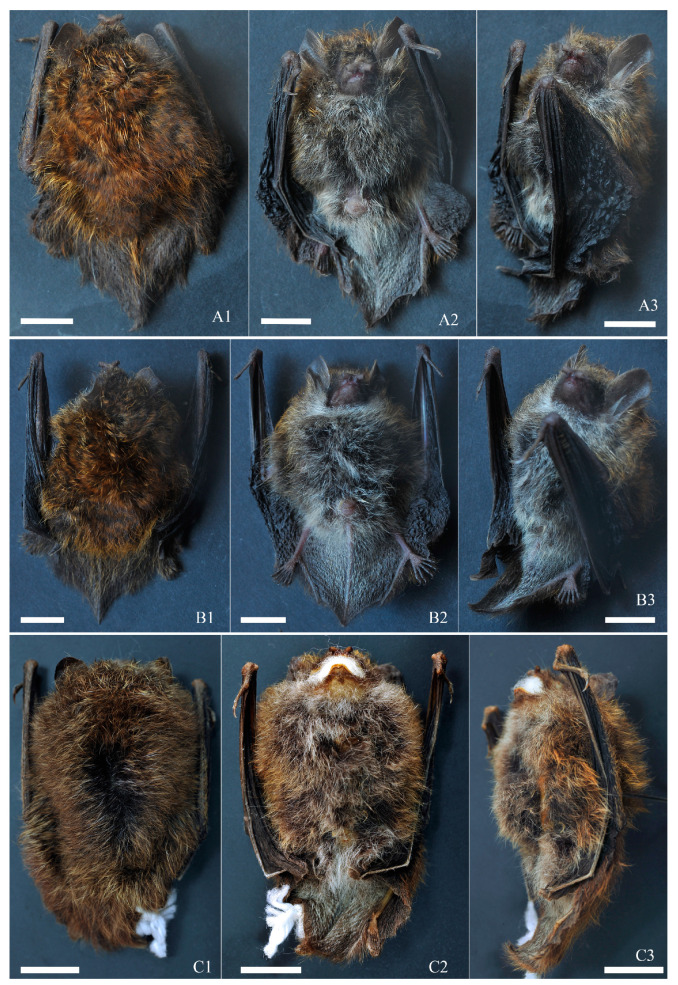
External morphology of *Murina yuanyang* sp. nov. (**A**) Holotype (KIZ20230424); (**B**) paratype (KIZ20230450); (**C**) paratype (KIZ20230415, specimens soaked in alcohol). (**1**) = Dorsal view; (**2**) = ventral view; (**3**) = lateral view. Scale = 10 mm.

**Figure 3:**
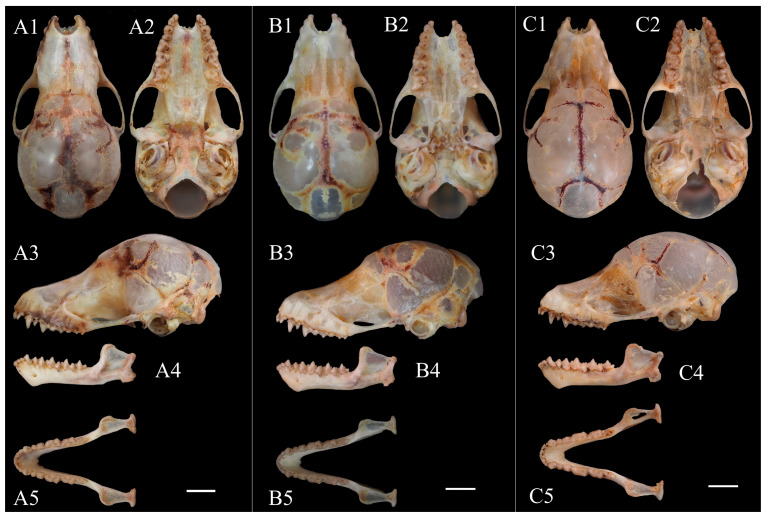
Skull of *Murina yuanyang* sp. nov. (**A**) Holotype (KIZ20230424); (**B**) paratype (KIZ20230450); (**C**) paratype (KIZ20230415). (**1**) = Dorsal view of skull; (**2**) = ventral view of skull; (**3**) = lateral view of skull; (**4**) = lateral view of mandible; (**5**) = occlusal view of mandible. Scale = 2 mm.

Diagnosis: Small-sized *Murina* species, FA 27.66–30.51 mm and GTL 13.44–14.16 mm (Table 4). Elongated tubular nostrils; third, fourth, and fifth finger metacarpals roughly equal in size; tail vertebrae slightly free at tip; plagiopatagium attached at about one-third from base of claw to base of toe, near base of claw (Figure 2). Fur on back glossy, overall dark golden with deep brown, with clearly demarcated color bands; ventral fur gray-white, with golden color on both sides of chest (Figure 2). Sagittal crest absent, lambdoidal crest not prominent (Figure 3); in lateral view, skull appears slightly elongated with slightly oval braincase, gradually rising with gentle slope from snout to cranial vertex (Figure 3(A3,B3)); palatine wide without distinct concavity (Figure 3(A2,B2,C2)). I^2^ located in front of I^3^, clearly visible from side; noticeable gap between I^3^ and C^1^; height of P^2^ less than half that of C^1^ and P^4^ (Figure 3(A3,B3,C3)); crown area of P^2^ slightly smaller than that of C^1^, less than half crown area of P^4^; mesostyle of M^1^ and M^2^ slightly developed, M^3^ reduced (Figure 3(A2,B2,C2)). C_1_ slightly higher than P_4_, P_2_ significantly lower than C_1_ and P_4_. Basal area of C_1_ similar to P_4_ and more than twice basal area of P_2_. Lower molars belong to nyctalodont type (Figure 3(A5,B5,C5)).

Description: Body: Small-sized *Murina* species, HB 31.37–36.34 mm, WT 3.4–4.7 g, FA 27.66–30.51 mm, GTL 13.44–14.16 mm (Table 4). Nostrils tubular, opening sideways and relatively long (Figure 2). Snout darker than nostrils. Ear length 11.85–14.77 mm, overall shape oval, upper part forms rounded arch, not pointed, deep gray in color, color at base slightly lighter than upper part, approaching gray-white. Ear tragus long and pointed, vertical on inside, curved outwards on outside, narrow at top and wider at bottom, reaching approximately half height of ear. Third, fourth, and fifth metacarpals approximately equal in length, second metacarpal slightly shorter than third, fourth, and fifth. Tail 23.89–31.58 mm (Table 4), shorter than head–body length, slightly free at tip (Figure 2). Plagiopatagium attached at about one-third from base of claw to base of toe, near base of claw (Figure 2).

Fur: Dorsal fur of holotype dark gold mixed with deep brown throughout, with four distinct bands of color on hairs. Base dark brown, middle light brown, upper-middle part with narrow dark brown or blackish brown band, top yellow-brown, sometimes golden yellow. Overall color of back appears slightly mottled and uneven. Short golden hairs on forearms and sparse yellow-brown hairs on interdigital membranes. Ventral side gray-white throughout, with base of hairs dark gray to gray and top gray-white. Neck and anal region whiter, with slight golden color on sides of chest. Relatively sparse gray-white hairs with slight sheen on interdigital membranes. Facial hair short, gray-brown mixed with golden yellow. Other specimens exhibit slight variations in dorsal fur color, appearing golden or yellowish, with distribution and characteristics of ventral and other fur similar to holotype (Figure 2).

Skull: Overall skull relatively small, GTL 13.44–14.16 mm. Sagittal crest absent, lambdoid crest prominent. In dorsal view, braincase almost circular; zygomatic arches weak and slender, gradually widening from front to back, with widest point at root of zygomatic arch; posterior margin of skull slightly prominent; slight downward concavity in middle from snout to frontal region. In lateral view, skull appears slightly elongated with slightly oval braincase; height from snout to parietal shows upward trend, with gradually increasing slope from snout to frontal, and gradually decreasing slope from frontal to parietal, resulting in slight depression between snout and frontal, with slight protrusion at frontal; zygomatic arch rises gradually from anterior to posterior. In ventral view, palatine wide without any obvious concavity, ending at midpoint of C^1^; basisphenoid pits tear-drop shaped, extending posteriorly to anterior half of cochlea. Mandible 8.09–8.69 mm. In lateral view, almost straight between coronoid process and condyle, without any depression, slight concavity between condyle and angle; angle short and wide, lower surface of the mandibular dentary forms obvious depression in front of angle; mental foramina clearly visible (Figure 3).

Dentition: Dental formula: $I\frac{-\;2\;3}{1\;2\;3}C\frac{1}{1}PM\frac{-\;2-\;4}{-\;2-\;4}M=34$. In maxilla, I^2^ positioned in front of I^3^, I^2^ clearly visible in lateral view; crown area of P^2^ slightly smaller than that of C^1^, less than half crown area of P^4^; species belongs to ‘s*uilla*-type’ based on these characteristics. Upper tooth rows converge slightly anteriorly, with PWC^1^C^1^ 1.42–1.78 mm and PWM^3^M^3^ 2.63–2.86 mm. I^2^ and I^3^ heights approximately equal, with crown area of I^2^ approximately half that of I^3^. I^2^ with two cusps, smaller secondary cusp located behind primary cusp. Posterior external face of I^2^ in contact with anterior internal face of I^3^, with noticeable gap between I^3^ and C^1^. C^1^ and P^4^ heights approximately equal, C^1^ slightly elongated and lacking secondary cusps, P^4^ wider. C^1^ appears slightly circular when viewed from occlusal perspective, with crown area less than half that of P^4^. P^2^ smaller and compressed, wider than long, distinctly oval-shaped, height close to half that of C^1^ and P^4^. Mesostyle of M^1^ and second upper molars (M^2^) not well developed; paracone, metacone, and protocone well developed, with metacone slightly higher than paracone; trigon basin open and talon well developed, with antero-external valley area significantly smaller than that of postero-external valley. M^3^ reduced, with only parastyle, paracone, and protocone. Single commissure connecting parastyle and paracone. In mandible, first, second and third lower incisors (I_1_, I_2_, and I_3_) tricuspid and of equal size, but outer cusp of I_3_ relatively less distinct; slight overlap of outer cusps of I_1_, I_2_, and I_3_; C_1_ contains pointed cusp on anterior inner margin, which touches outer cusp of I_3_, making C_1_ slightly higher than I_3_ in lateral view, with gradual increase in height from I_1_ to C_1_. C_1_ slightly higher than P_4_, P_2_ significantly lower than C_1_ and P_4_. Basal area of C_1_ similar to P_4_ and more than twice basal area of P_2_. In lateral view, trigonid of M_1_ and lower second molar (M_2_) and M_3_ clearly tricuspid, with height of metaconid and paraconid approximately two-thirds that of protoconid; talonid of M_1_ and M_2_ bicuspid, with entoconid and hypoconid clearly separated from trigonid and lower than metaconid and paraconid, with heights approximately equal to metaconid and paraconid. Talonid of M_3_ reduced. Lower molars nyctalodont type, with entoconid and hypoconid connected by postcristid (Figure 3).

Comparisons: Based on its dentition, *Murina yuanyang* sp. nov. clearly belongs to the ‘*suilla*-type’ (maxillary toothrows clearly convergent anteriorly; I^2^ anterior to I^3^; I^2^ clearly visible from lateral view; crown area of P^2^ half that of P^4^) and can be distinguished from all species in the ‘c*yclotis*-type’. Based on the reconstructed phylogenetic tree, *Murina yuanyang* sp. nov. formed a monophyletic group with *M. chrysochaetes* and was distantly related to other species. Therefore, our comparison focuses primarily on species closely related in the phylogenetic tree and on ‘*suilla*-type’ species lacking COI sequences.

**Figure 4:**
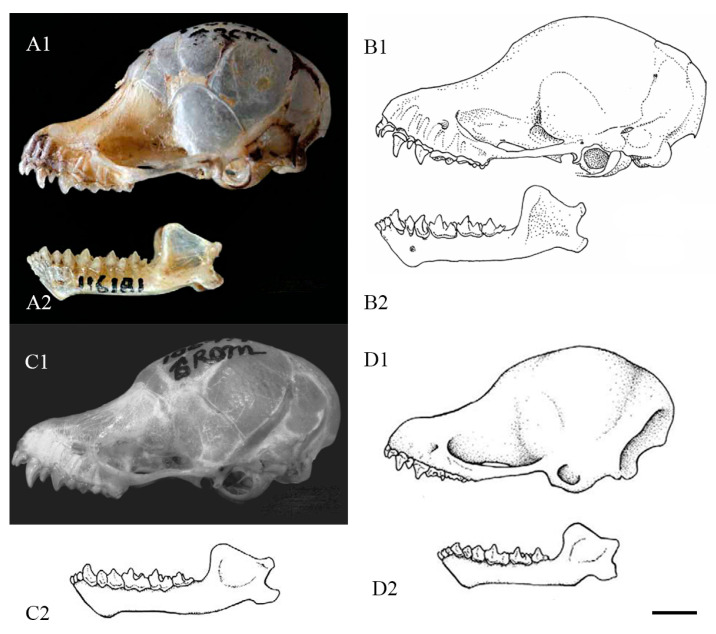
Lateral view of skulls from principal species within the *Murina* genus for comparative analysis, sourced from the literature. (**A**) *M. chrysochaetes* [18]; (**B**) *M. harpioloides* [17]; (**C**) *M. eleryi* [1,7]; (**D**) *M. aurata* [7]. (**1**) = maxilla; (**2**) = mandible. Scale = 2 mm.

Comparison with *M. chrysochaetes*: At the molecular level, *Murina yuanyang* sp. nov. is most closely related to *M. chrysochaetes*, but exhibits some differences in morphology and cranial structure. Measurement data for FA and TIB show that *Murina yuanyang* sp. nov. is slightly larger in body size than *M. chrysochaetes*, and the HB, TL, E, and HF of the female specimens also showed longer HF and E and significantly different HB and TL ratios (HB/TL ratios were 1.23 and 1.67, respectively) compared with IEBR-M6020 and S186699 (both female). In terms of skull size, both male and female *Murina yuanyang* sp. nov. are slightly smaller than *Murina chrysochaetes* (values of STOTL, GTL, CBL, ZYW, BCW). From PWC^1^C^1^/PWM^3^M^3^ and C^1^C^1^W/M^3^M^3^W, the values of male *Murina yuanyang* sp. nov. are 0.59 and 0.67, respectively, and female are 0.60 and 0.69, respectively, while the values of S186699 (female) are 0.66 and 0.74, respectively, which is consistent with the observation from the specimen comparison that the maxillary teeth of *Murina yuanyang* sp. nov. converge more anteriorly than the holotype of *Murina chrysochaetes*. The value of CPH shows that *Murina chrysochaetes* has a higher coronoid process. In terms of external morphology, *Murina yuanyang* sp. nov. has longer tubular nostrils. On the dorsal side, *Murina yuanyang* sp. nov. is generally dark gold mixed with deep brown, with four color bands on the back, while *M. chrysochaetes* has a mixture of black and gold stripes and tricolored fur on the back [18]. On the ventral side, *Murina yuanyang* sp. nov. shows a golden hue on the sides of the thorax, while *M. chrysochaetes* has golden guard hairs from the thorax to lower abdomen, giving it an overall golden appearance. The wing and tail membranes of *Murina yuanyang* sp. nov. are black, in contrast to those of *M. chrysochaetes*, which are brown. Regarding the skull structure, the rostrum of *Murina yuanyang* sp. nov. is relatively long, with a gentler forehead slope, resulting in a more elliptical skull shape in lateral view. In contrast, the rostrum of *M. chrysochaetes* is short and the forehead slope is abrupt, producing a round shape in lateral view. The zygomatic arch of *Murina yuanyang* sp. nov. is thicker and smoother compared to that of *M. chrysochaetes*, which is thinner and shows a slight concavity. In the mandible, *Murina yuanyang* sp. nov. has a wider and more robust angle. C_1_ of *Murina yuanyang* sp. nov. slightly higher than P_4_ and basal area of C_1_ similar to P_4_, while C_1_ of *M. chrysochaetes* is the same height as P_4_ but exceeds it in basal area. Furthermore, C^1^ of *M. chrysochaetes* is recurved, whereas C^1^ of *Murina yuanyang* sp. nov. is straighter, with the talons of M^1^ and M^2^ more developed (Figure 3 and Figure 4A).

Comparison with *M. harpioloides*: *Murina yuanyang* sp. nov. and *M. harpioloides* show similarities in morphometric data, including WT, HB, TL, and FA; however, the skull and tooth measurements of *Murina yuanyang* sp. nov. are slightly smaller than those of *M. harpioloides* (Table 4). Notably, in the ventral view of the skull, the talons of M^1^ and M^2^ of *Murina yuanyang* sp. nov. are more developed, and M^3^ is slightly fuller with a different angle compared to *M. harpioloides*. The curvature at the posterior part of the pterygoid differs significantly between the two species. In the occlusal view of the mandible, P_4_ of *Murina yuanyang* sp. nov. is more elliptical, M_1_ and M_2_ are slenderer, and the talonid appears sharper; the angle formed by the paracristid and protocristid of M_2_ is larger than that of *M. harpioloides*. From the lingual view, C_1_ of *M. harpioloides* is more pointed and narrows more obviously in the middle (Figure 3 and Figure 4B).

Comparison with *M. eleryi* and *M. aurata*: Eger and Lim [18] identified *M. chrysochaetes* as most similar in size and appearance to *M. eleryi* and *M. aurata*, although with a smaller body size. *Murina yuanyang* sp. nov. is slightly larger than *M. chrysochaetes*, but still smaller than *M. eleryi* and *M. aurata*, with comparatively smaller skull measurements (Table 4). Furey et al. [7] distinguished *M. eleryi* from *M. aurata* based on larger and longer canines, a feature that also differentiates *Murina yuanyang* sp. nov. from *M. eleryi*. In addition, the mesostyles on M^1^ and M^2^ are much less developed in *Murina yuanyang* sp. nov. than *M. eleryi*. Male *Murina yuanyang* sp. nov. specimens also have longer CM^3^L and CM_3_L compared to the male holotype and paratype of *M. aurata*, while GTL, CBL, and MDL measurements are significantly shorter, indicating a larger ratio of maxillary and mandibular canine–molar length relative to skull length in *Murina yuanyang* sp. nov. Furthermore, *Murina yuanyang* sp. nov. can be distinguished from *M. aurata* by the relative positions of I^2^ and I^3^, development of the tooth bases, shape of P^2^, and width of the gap between P^2^ and P^4^. Morphologically, *Murina yuanyang* sp. nov. has longer tubular nostrils and dark golden fur with brown stripes, differing from the golden fur of *M. aurata* described by Milne-Edwards [32] and the varying yellow-brown to gray-brown to copper-red shades of *M. eleryi* described by Furey [7] (Figure 3 and Figure 4C,D).

Comparison with other ‘s*uilla*-type’ species without COI sequences: Other ‘s*uilla*-type’ species lacking COI sequences include *M. beelzebub*, *M. bicolor*, *M. fanjingshanensis*, *M. ryukyuana*, and *M. tenebrosa*. According to Maeda and Matsumura [33], Kuo et al. [8], Csorba et al. [6], and He et al. [20], these species are classified as medium- to large-sized within the *Murina* genus (Table 5), distinguishing them from the smaller-sized *Murina yuanyang* sp. nov.

Distribution and habitat: The *Murina yuanyang* sp. nov. specimens were captured at two different locations in Pinghe, Xiaoxin Street, Yuanyang County, Yunnan Province, China (22.99° N, 103.00° E and 22.99° N, 102.99° E). The habitat consists of a mid-mountain evergreen broad-leaved forest at elevations of 2412 m and 2434 m, respectively. The area features a well-closed canopy, abundant shrubs, and several small streams. Although there are no known caves in the area, the forest contains many large trees with hollows. It is suspected that this insectivorous bat primarily roosts during the day in tree hollows or beneath the dense canopy.

## 4. Discussion

We conducted an in-depth analysis of these seven unique specimens using both morphological and molecular approaches.

At the molecular level, we sequenced the COI gene and the mitochondrial cytochrome B (Cyt b) gene in some specimens. Since the holotype specimen of *M. chrysochaetes*, which is the closest relative of *Murina yuanyang* sp. nov., has only COI sequences, and there are more COI sequences than Cyt b available for species of the genus *Murina*, we finally chose the COI sequence for molecular analysis. We used these sequences to calculate the uncorrected *p*-distances among species and reconstruct their phylogenetic relationships. In the resulting phylogenetic tree, all *Murina yuanyang* sp. nov. formed a clade and a distinct lineage sister to *M. chrysochaetes* with a posterior probability of 1 and a bootstrap value of 100, respectively, indicating that *Murina yuanyang* sp. nov. and *Murina chrysochaetes* have a differentiated but close phylogenetic relationship. However, the uncorrected *p*-distance between *Murina yuanyang* sp. nov. and *M. chrysochaetes* was 2.7–3.0%, a value that is only greater than that between *M. leucogaster* and *M. shuipuensis* (2.6%) (Table 2). Avise [34] found that intraspecific genetic differentiation is typically less than 2%, with most cases falling below 1%. Hebert et al. [35] reported that over 98% of species pairs exhibit more than 2% sequence divergence, and later suggested that the standard threshold for species divergence should be 10 times the average genetic distance within a species [36]. In this study, the minimum genetic distance between *Murina yuanyang* sp. nov. and *M. chrysochaetes* was 2.7%, significantly higher than 10 times the average intraspecific genetic distance of 0.1% for *Murina yuanyang* sp. nov. This distance also exceeds the 2% divergence threshold described by Hebert et al. [35].

Morphologically, we described and compared *Murina yuanyang* sp. nov. with two genetically close species, *M. chrysochaetes* and *M. harpioloides*, and two morphologically similar species, *M. eleryi* and *M. aurata*, as well as other members of the ‘s*uilla*-type’ lacking COI sequences. *Murina yuanyang* sp. nov. can be distinguished from *M. beelzebub*, *M. bicolor*, *M. fanjingshanensis*, *M. ryukyuana*, and *M. tenebrosa* by body size; from *M. eleryi* by skull size, C^1^, development of the mesostyles on M^1^ and M^2^, and fur color; from *M. aurata* by fur color, skull size, ratio of maxillary and mandibular canine–molar length relative to skull length, the relative positions of I^2^ and I^3^, development of the tooth bases, shape of P^2^, and width of the gap between P^2^ and P^4^; from *M. harpioloides* by skull and tooth size and tooth structure; and from *M. chrysochaetes* by tubular nares, fur on the back and abdomen, rostrum, the slope of the forehead, zygomatic arches, C^1^, height and base area of C_1_ and P_4_, and measurements. The other species were not compared morphologically in detail, either because they belong to the *cyclotis*-type species or because they are far away from the *p*-distance of *Murina yuanyang* sp. nov. Given the widespread sexual dimorphism in the subfamily Murininae, we used the same-sex specimens when comparing the *Murina yuanyang* sp. nov. with the above species in the Comparison section. The sexual dimorphism of *Murina yuanyang* sp. nov. is mainly manifested in the external and cranial dimensions, the slope of the forehead, and the shape of P^2^.

Although *Murina yuanyang* sp. nov. and *M. chrysochaetes* can be clearly distinguished by morphological characteristics, and there are indeed valid species with a small genetic distance in the *Murina*, the reason for the small genetic distance between them deserves further study. For sister taxa *M. recondita* and *M. gracilis*, Kuo et al. [37] confirmed that the introgression of mitochondrial DNA did exist in the two species in history by studying their differentiation history (this introgression has ceased at present), and explained the possible reasons for this phenomenon, which is of great reference value for the situation between *Murina yuanyang* sp. nov. and *M. chrysochaetes*. To determine whether there is an introgression of mitochondrial DNA between *Murina yuanyang* sp. nov. and *M. chrysochaetes*, as well as their differentiation history and population dynamics, an in-depth study needs to be conducted. This will require more experimental material.

## 5. Conclusions

A new species of the *Murina*, *Murina yuanyang* sp. nov., is described in this paper, based on seven specimens collected from the Guanyinshan Provincial Nature Reserve, Yuanyang, Yunnan, China. Currently, the new species is known only from its type locality. The local ecological environment is relatively well maintained [38], and this species is less threatened at present.

## Figures and Tables

**Table 1:** List of cranial and dental measurements used in this study.

Character	Definitions
STOTL	total length of skull: from anterior rim of alveolus of I^2^ to most projecting point of occipital region
GTL	greatest length of skull: from anterior aspect of I^2^ to most prominent point of occipital region
CBL	condylobasal length: from exoccipital condyle to posterior rim of alveolus of I^2^
CCL	condylocanine length: from exoccipital condyle to most anterior part of C^1^
ZYW	zygomatic width: greatest width of skull across zygomatic arches
BCW	braincase width: greatest width of braincase
BCH	braincase height: skull placed horizontally, measuring from horizontal plane to highest point of cranium
IOW	interorbital width: least width of interorbital constriction
POW	width of postorbital constriction: least width of postorbital constriction
MAW	mastoid width: greatest distance across mastoid region
BOW	basioccipital width: least distance between cochleae
CM^3^L	upper canine–molar length: from anterior of C^1^ to posterior of M^3^ crown
IM^3^L	maxillary toothrow length: from anterior point of I^2^ to back of M^3^ crown
CP^4^L	upper canine–premolar length: from anterior of C^1^ to posterior of P^4^ crown
M^1^M^3^L	upper molar length: from anterior of first upper molar (M^1^) to posterior of M^3^
PWC^1^C^1^	anterior palatal width: least distance between inner borders of C^1^
PWM^3^M^3^	posterior palatal width: least distance between inner borders of M^3^
C^1^C^1^W	upper canine width: greatest width of outer borders of C^1^
M^3^M^3^W	upper molar greatest width: greatest width of outer borders of M^3^
MDL	greatest length of mandible: from anterior point of first lower incisor (I^2^) to most posterior part of condyle
ML	mandible length: from anterior rim of alveolus of I^2^ to most posterior part of condyle
CPH	coronoid process height: least distance from apex of coronoid process to indentation of lower border of ramus mandibula
CM_3_L	lower canine–molar length: from anterior of C_1_ to posterior of third lower molar (M_3_) crown
IM_3_L	mandibular toothrow length: from anterior point of I_2_ to back of M_3_ crown
CP_4_L	lower canine–premolar length: from anterior of lower canine to back of posterior P_4_ crown
M_1_M_3_L	lower molar length: from anterior of first lower molar (M_1_) to the posterior of M_3_.

**Table 2:** Species and GenBank accession numbers of sequences used in phylogenetic reconstruction.

Taxon	Accession Number	Taxon	Accession Number
*Harpiocephalus harpia*	HM540274	*Murina huttoni*	JQ601452
*Harpiola isodon*	HM540286	*Murina jaintiana*	MF537346
*Kerivoula kachinensis*	MZ438743	*Murina jinchui*	MN549070
*Murina aenea*	HM540928	*Murina kontumensis*	KT820760
*Murina annamitica*	HM540969	*Murina leucogaster*	HM540988
*Murina balaensis*	KY034093	*Murina lorelieae*	JN082179
*Murina chrysochaetes*	HM540986	*Murina peninsularis*	HM540972
*Murina cyclotis*	JF443973	*Murina puta*	KT982277
*Murina eleryi*	KT762293	*Murina recondita*	KJ198687
*Murina feae*	KF772778	*Murina rongjiangensis*	MN549085
*Murina fionae*	HM540965	*Murina rozendaali*	KY034110
*Murina gracilis*	KJ198567	*Murina shuipuensis*	JN082180
*Murina guilleni*	KY034137	*Murina suilla*	KY034090
*Murina harpioloides*	JF443974	*Murina tubinaris*	HM541000
*Murina harrisoni*	MN549045	*Murina ussuriensis*	HQ974648
*Murina hilgendorfi*	JF442833	*Murina walstoni*	HM540957
*Murina hkakaboraziensis*	MF537343	*Myotis muricola*	MW054913

**Table 3:** Uncorrected *p*-distances (%) between species calculated from 657 bp COI gene fragment. Values less than or equal to *p*-distances between our specimen and *M. chrysochaetes* are underlined.

No.	Taxon	1	2	3	4	5	6	7	8	9	10	11	12	13	14
1	*M. yuanyang* sp. Nov. (KIZ20230415)														
2	*M. yuanyang* sp. Nov. (KIZ20230424)	0.0													
3	*M. yuanyang* sp. Nov. (KIZ20230426)	0.0	0.0												
4	*M. yuanyang* sp. Nov. (KIZ20230449)	0.0	0.0	0.0											
5	*M. yuanyang* sp. Nov. (KIZ20230492)	0.0	0.0	0.0	0.0										
6	*M. yuanyang* sp. Nov. (KIZ20230498)	0.3	0.3	0.3	0.3	0.3									
7	*M. chrysochaetes*	2.7	2.7	2.7	2.7	2.7	3.0								
8	*M. harpioloides*	9.0	9.0	9.0	9.0	9.0	9.3	9.4							
9	*M. eleryi*	16.3	16.3	16.3	16.3	16.3	16.3	17.2	16.5						
10	*M. leucogaster*	17.0	17.0	17.0	17.0	17.0	17.4	16.9	16.0	16.6					
11	*M. rongjiangensis*	16.6	16.6	16.6	16.6	16.6	16.9	17.0	16.0	15.7	4.3				
12	*M. shuipuensis*	17.5	17.5	17.5	17.5	17.5	17.8	17.7	16.4	16.1	2.6	4.1			
13	*M. gracilis*	17.8	17.8	17.8	17.8	17.8	17.8	18.8	17.5	9.6	17.5	16.6	17.0		
14	*M. recondita*	19.1	19.1	19.1	19.1	19.1	19.1	20.0	18.6	10.7	18.4	17.2	17.5	4.2	

**Table 4:** Weight (g) and external and cranial measurements (mm) of *Murina* species. Abbreviations and definitions of measurement values are provided in Section 2. “♂” indicate male, “♀” indicate female, “*” indicate the means of IEBR-M6020 and S186699.

Character	*Murina yuanyang* sp. nov.				*Murina chrysochaetes*		*Murina harpioloides* Holotype, ♀, Kruskop and Eger [17]	*Murina eleryi* Holotype, ♂, Furey et al. [7]	*Murina aurata* Holotype, ♂ Paratype, ♂ Mean (*n* = 2) Eger and Lim [18]
	Holotype (KIZ20230424)	Paratype (KIZ20230450)	♂ (*n* = 2) Mean, KIZ20230415 (Paratype), KIZ20230492	♀ (*n* = 5) Mean ± SD, Min–Max	Holotype, ♂, Eger and Lim [18]	IEBR-M6020, ♀/S186699, ♀, Son et al. [10,31]			
WT	4.2	4.7	3.5, 3.6, 3.4	4.3 ± 0.3, 3.8–4.7	3.0	4.0 *	4.2	4.0	-
HB	35.05	36.34	33.66, 31.37, 35.94	34.82 ± 1.52, 32.11–36.34	-	40.0 *	35.0	-	-
TL	31.58	27.99	25.57, 23.89, 27.25	28.39 ± 1.64, 26.92–31.58	-	24.0 *	30.5	28.7	-
E	12.99	13.11	13.09, 12.77, 13.40	13.25 ± 0.82, 12.27–14.77	-	12.0 *	12.3	12.6	-
HF	7.88	8.52	6.49, 6.83, 6.14	7.66 ± 0.75, 6.25–8.52	-	5.5 *	-	6.2	-
TIB	13.48	13.57	12.86, 12.56, 13.16	13.15 ± 0.43, 12.37–13.57	10.92	12.6 *	-	14.7	12.00
FA	29.8	29.64	27.81, 27.96, 27.66	29.83 ± 0.35, 29.53–30.51	26.35	28.6 *	29.7	28.4	29.14
MET2	25.56	25.55	22.36, 22.82, 21.89	24.46 ± 1.00, 23.06–25.56	-	-	-	-	-
MET3	27.90	28.09	25.15, 24.48, 25.79	27.39 ± 0.60, 26.58–28.09	25.43	-	-	26.2	25.99
MET4	27.18	27.79	25.09, 24.59, 25.59	26.99 ± 0.68, 25.78–27.79	24.53	-	-	25.5	26.46
MET5	27.49	27.46	25.34, 24.95, 25.73	27.21 ± 0.28, 26.71–27.49	24.50	-	-	26.1	26.81
STOTL	13.84	13.79	13.54, 13.31, 13.77	13.90 ± 0.10, 13.79–14.07	-	14.57/14.72	-	14.62	-
GTL	14.13	13.95	13.72, 13.44, 14.00	14.09 ± 0.07, 13.95–14.16	14.05	-	-	14.90	14.20
CBL	12.68	12.46	12.18, 12.02,12.35	12.58 ± 0.12, 12.41–12.72	12.45	-	13.02	12.89	12.56
CCL	12.12	12.05	11.75, 11.58, 11.93	12.10 ± 0.09, 11.94–12.22	-	-	12.34	12.59	-
ZYW	7.73	7.70	7.46, 7.41,7.52	7.69 ± 0.13, 7.44–7.81	7.85	8.41	-	7.84	7.59
BCW	6.95	6.97	6.92, 6.91, 6.93	7.00 ± 0.08, 6.93–7.15	6.98	7.28	7.21	7.11	7.15
BCH	6.78	6.56	6.79, 6.59, 6.98	6.62 ± 0.09, 6.51–6.78	-	6.33	5.81	5.77	-
IOW	3.84	3.80	3.73, 3.67, 3.78	3.79 ± 0.06, 3.68–3.86	-	4.17	4.12	4.27	-
POW	3.93	3.90	3.91, 3.88, 3.93	3.94 ± 0.05, 3.88–4.01	4.26	-	4.09	-	4.17
MAW	7.38	7.38	7.21, 7.18, 7.23	7.31 ± 0.10, 7.12–7.38	7.07	7.62	7.42	7.07	7.14
BOW	1.50	1.38	1.32, 1.28, 1.35	1.40 ± 0.06, 1.30–1.50	-	1.39	-	1.22	-
CM^3^L	4.51	4.54	4.41, 4.43, 4.38	4.53 ± 0.06, 4.45–4.63	4.36	5.44/4.66	4.68	4.50	4.27
IM^3^L	5.28	5.29	5.18, 5.13, 5.23	5.28 ± 0.07, 5.22–5.40	-	-	-	-	-
CP^4^L	2.00	2.11	2.03, 2.06,2.00	2.07 ± 0.04, 2.00–2.12	-	2.02	-	2.07	-
M^1^M^3^L	2.73	2.68	2.67, 2.63, 2.71	2.69 ± 0.04, 2.62–2.74	-	2.88	-	-	-
PWC^1^C^1^	1.78	1.71	1.53, 1.42, 1.63	1.68 ± 0.07, 1.55–1.78	-	1.99	-	1.65	-
PWM^3^M^3^	2.73	2.76	2.69, 2.63, 2.75	2.80 ± 0.05, 2.73–2.86	-	3.01	3.10	2.59	-
C^1^C^1^W	3.22	3.28	3.11, 3.01, 3.21	3.21 ± 0.08, 3.06–3.28	3.18	3.63	3.39	3.21	3.18
M^3^M^3^W	4.74	4.62	4.60, 4.61, 4.59	4.67 ± 0.04, 4.62–4.74	-	4.93	4.88	4.62	-
MDL	8.76	8.75	8.32, 8.21, 8.43	8.75 ± 0.13, 8.51–8.91	8.42	-	-	-	8.59
ML	8.41	8.42	8.12, 8.09, 8.15	8.49 ± 0.13, 8.34–8.69	-	9.30/9.94	9.31	9.55	-
CPH	2.86	2.93	2.64, 2.52, 2.76	2.83 ± 0.09, 2.69–2.93	3.06	3.51	3.32	2.86	2.87
CM_3_L	5.03	4.93	4.76, 4.81, 4.71	4.93 ± 0.05, 4.88–5.03	4.36	5.08	5.13	4.89	4.64
IM_3_L	5.48	5.56	5.34, 5.32, 5.36	5.57 ± 0.09, 5.46–5.70	-	-	-	-	-
CP_4_L	2.01	1.92	1.88, 1.85, 1.91	1.92 ± 0.06, 1.85–2.01	-	1.83	-	1.82	-
M_1_M_3_L	3.34	3.42	3.20, 3.16, 3.24	3.36 ± 0.05, 3.29–3.42	-	3.33	-	-	-

**Table 5:** Comparison of body size of *Murina yuanyang* sp. nov. and other ‘s*uilla*-type’ species lacking COI sequences.

	Size	FA	STOTL	GTL
*Murina yuanyang* sp. nov. (*n* = 7)	Small	27.66–30.51	13.31–14.07	13.44–14.16
*M. beelzebub*, holotype Csorba et al. [6]	Medium	33.7	16.54	
*M. bicolor* Kuo et al. [8] (*n* = 8)	Large	37.2–41.6	-	18.00–19.54
*M. fanjingshanensis* He et al. [20] (*n* = 3)	Large	40.60–41.44	-	18.39–19.05
*M. ryukyuana* Maeda and Matsumura [32] (*n* = 4)	Medium	35.5–37.0	-	18.30–18.65
*M. tenebrosa* Kuo et al. [8]	Medium	33.8	-	16.81

## Data Availability

All data are presented in this article.
